# Exploring the concept of non-violent resistance amongst healthcare workers

**DOI:** 10.1177/09697330221122904

**Published:** 2022-10-06

**Authors:** Ryan Essex, Hil Aked, Rebecca Daniels, Paul Newton, Sharon Weldon

**Affiliations:** Institute for Lifecourse Development, 4918 University of Greenwich, London, UK; 33373 Medact, London, UK; Institute for Lifecourse Development, 4918 University of Greenwich, London, UK

**Keywords:** protest, resistance, non-violent resistance, healthcare, healthcare workers, ethics

## Abstract

**Background:**

Non-violent resistance which has involved healthcare workers has been instrumental in securing a number of health-related gains and a force in opposing threats to health. Despite this, we know little about healthcare workers who have engaged in acts of non-violent resistance.

**Research aim:**

Amongst a sample of healthcare workers who had engaged in acts of resistance this study sought to explore their understanding of non-violent resistance and how or whether they felt healthcare workers made a distinct contribution to such action.

**Research design:**

Cross-sectional survey

**Participants and research context:**

Healthcare workers (doctors, nurses, academics and others) from the UK and Europe who had engaged in acts of non-violent resistance.

**Ethical considerations:**

Ethical approval for this study was granted by the University of Greenwich Human Research Ethics Committee (UREC/20.5.6.11).

**Findings/results:**

Most participants spoke about the nature of non-violent resistance, its oppositional, didactic and symbolic functions and the role of violence or harm. While most people understood non-violent resistance as a public, oppositional and collective act, many identified more subtle everyday acts in the workplace that undermined policy or procedures they saw as harmful. When asked about distinctions in non-violent resistance carried out by healthcare workers, most participants referred to their standing in society, noting that healthcare works were a trusted and authoritative source. Some identified an ethical imperative to act while others identified the risks that came with such action, noting their accountability and responsibility they had to patients. About a quarter of participants felt that such action was no different to others carrying out non-violent resistance or dependent on the issue or nature of the action.

**Conclusions:**

These findings speak to the complex and multifaceted nature of non-violent resistance. Additionally our findings suggest healthcare workers have a distinct role to play in leading and supporting non-violent actions.

## Introduction

Resistance has been instrumental in securing a number of health-related gains and an important force in opposing threats to health and wellbeing.^1^ We can see the impact of agitation and opposition as early as the 1800s in achieving improved sanitation^2^ and access to birth control.^3^ We need not look far to find a range of more contemporary examples; the COVID-19 pandemic has more recently prompted significant unrest related to health. Globally there has been protests from healthcare workers, related to a lack of personal protective equipment (PPE) and inadequate government responses to the pandemic.^4,5^ There have also been protests against public health measures, such as vaccines, lock downs and face coverings, many also involving healthcare workers.^6^ At the same time, and during the pandemic, we have seen protests from Doctors for Extinction Rebellion. On the 3rd of September 2021, 60 doctors in London, nurses and other health professionals staged a die-in protest outside JP Morgan’s headquarters in London to highlight the bank’s investment in fossil fuels. Private security guards swiftly removed the protesters who sat or lay on the pavement. The die-in was staged to symbolise the deaths caused by fossil fuel investment.^7^ While we can easily find a number of public examples of such action, resistance is far more ubiquitous than just public acts. Overlooked are the everyday acts that undermine, degrade or even sabotage unfair systems and structures. Such acts are more difficult to quantify, as in many cases they are meant to be clandestine. However, we can also begin to find examples in the literature, for instance, medical students challenging senior clinicians and hierarchy^8^ and in how nursing students negotiated resistance while on placement.^9^ A recent body of work has examined how Swedish General Practitioners resisted in relation to providing sickness certificates provided for the Swedish Social Insurance Agency.^10^ Despite the numerous examples and a growing body of literature, non-violent resistance remains a contested concept; this study sought to explore this with a sample of healthcare workers who had themselves engaged in non-violent actions.

### Background

Given that resistance could take so many forms, it is perhaps unsurprising that the terms non-violent resistance and resistance have been described as having a ‘palpable lack of definitional consensus’.^11^ These terms also sit alongside a range of similar terms: ‘critical resistance’, ‘off-kilter resistance’, ‘civil resistance’, ‘non-violence resistance’^12^ and ‘dispersed resistance’.^13^ To further complicate this picture, non-violent resistance is often used interchangeably with a range of terms such as ‘activism’, ‘contentious politics’, ‘protest’ and ‘civil disobedience’. Perhaps the greatest points of contention however rest with the fact that resistance can be so expansive, something which we touched upon briefly above. There has been much discussions in recent decades about what could be best termed everyday acts of resistance.^14^ This type of resistance has generally been contrasted to more open collective acts of resistance, as hidden and individual, not seeking to directly confront power. Definitions which encompass this type of action have defined resistance as ‘any action imbued with intent that attempts to challenge, change or retain particular circumstances relating to societal relations, processes and/or institutions… [which] imply some form of contestation… [and] cannot be separated from practices of domination’,^15^ as ‘a broad range of dissident activities, of varying scope and impact, which express opposition, and perhaps refusal to conform, to a dominant system of values, norms, rules, and practices’^16^ and as ‘any act, performed by any individual (or collective) … that is a response to power, most often in opposition to contentious, harmful or unjust rules, practices, policies or structures’.^17^ Perhaps unsurprisingly then, discussion continues about what resistance is, with particular contention related to the nature of intent,^18^ the often binary conceptualisation of everyday and other forms of resistance^19^ and its relationship to power. Some have suggested that rather than simply being oppositional, resistance is entangled with power. Vinthagen and Johansson,^20^ for example, suggest that agents can be both the subject and object of power and argue that ‘[a]gents of resistance often simultaneously promote power-loaded discourses, being the bearers of hierarchies and stereotypes as well as of change’.

While throughout the nursing and broader health literature we can begin to find examples of resistance and while there is a relatively large scholarship that exists related to power, the healthcare professions and healthcare, including nursing,^21^ there remains little empirical research in this area. This has not only left many questions unanswered related to the normative and regulatory issues resistance raises, but conceptually, what resistance is and its relationship to health and healthcare. To our knowledge, these issues have not been explored with healthcare workers more generally and those who have engaged in acts of resistance. This exploratory study sought to address this gap exploring these fundamental questions amongst a sample of healthcare workers who have engaged in acts of resistance, specifically this study sought to explore their understanding of non-violent resistance and how or whether they felt healthcare workers made a distinct contribution to such action.

## Methods

### Procedure and participants

Participants were recruited online via a database of approximately 7000 healthcare workers and academics, both retired and still working and mainly based in the UK and Europe. Participants were recruited through the Medact (https://www.medact.org/) member database. An email was sent to potential participants explaining the purpose of the study and a link where participants were directed to an online survey hosted on Qualtrics. Medact is non-profit charity that has a membership primarily made up of healthcare workers, organising around the production of research and campaigning, with a primary focus on peace and security, climate and health, economic justice and health and human rights.

### Survey and data collection

The survey included open and closed questions related to participant demographics and their involvement in non-violent actions. The survey was open for 3 months and responses were collected between July and September 2021. The results here focus on questions that explored participants’ understanding of non-violent resistance and whether they felt it was unique or distinct when carried out by healthcare workers. More specifically, in addition to asking several questions about participant demographics, profession and their involvement in non-violent action, participants were asked how they would define non-violent resistance/action and whether the involvement of healthcare workers in such action made it distinct or different in any way.

### Analysis

To analyse sample characteristics and their engagement in non-violent resistance, descriptive analyses were carried out in SPSS 27.^22^ Content analysis was used to explore open responses, this technique identifies the number of times a theme or issue is present in participants’ responses and then presenting this utilising descriptive statistics and a narrative.^23^ The steps involved in qualitative content analysis, as described by White and Marsh^23^ were followed. Namely, the research question was formulated and coding was carried out with analysis integrated. Coding involved reading through all responses to identify key phrases and segments of text, noting places of convergence and divergence. This continued iteratively, until categories and sub-categories emerged. Initial coding was carried out by RE which resulted in a draft coding scheme. All authors were then involved in subsequent revision of this scheme, again employing an iterative approach until all authors agreed upon categories and sub-categories.

### Ethical approval

Ethical approval for this study was granted by the University of Greenwich Human Research Ethics Committee (UREC/20.5.6.11). Participants were informed of the purpose of the study, all were informed that participation was voluntary and that they could withdraw from the survey at any time.

## Results

### Sample characteristics and engagement in non-violent resistance

Overall there were 148 responses to the survey; 55 (37%) respondents were retired. The majority of participants who responded to the survey were doctors (*n* = 77, 52%), a much smaller number reported they were nurses (*n* = 13, 8.8%) and academics (*n* = 8, 5.4%), the remainder of participants came from a variety of health backgrounds. Almost all participants indicated they had engaged in non-violent resistance (*n* = 128; 87%). Three participants indicated they had not engaged in such action, while 17 left this question blank. The majority of participants (*n* = 81; 61%) indicated they engaged in non-violent resistance regularly, at least a few times a year. The most common forms of action were marches (*n* = 118, 79.7%), online activities (*n* = 104, 70.3%), lobbying (*n* = 80, 54.1%) and flyering/leafleting (*n* = 70, 47.3%). The activities least engaged in included whistleblowing (*n* = 25, 16.9%) and sit-ins (*n* = 34, 23%).

### Understandings of non-violent resistance

**Figure 1.**
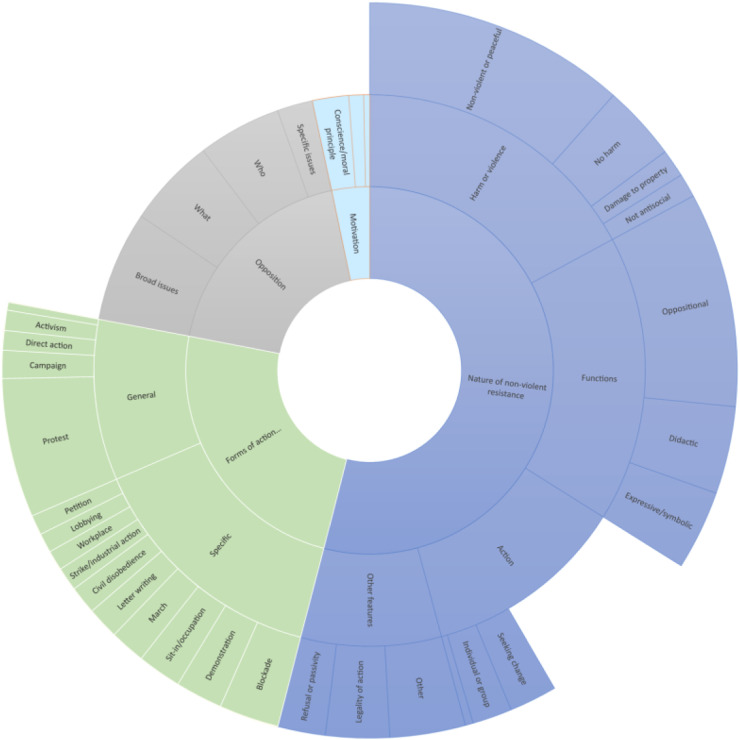
Sunburst chart of responses when asked about the nature of non-violent resistance. Note: This chart represents all responses to the question about the nature of non-violent resistance. The overarching themes are in the centre of the circle, with sub-themes found further out. The space occupied by each theme represents the number of participants who mentioned it. For example, in this figure, a little more than half of the participants spoke about the nature of resistance itself, with most mentioning the role of violence or harm in such action and its functions, namely, its oppositional, didactic and expressive/symbolic functions.

Participants were asked to elaborate on their understanding of non-violent resistance. Overall there were 140 responses to this question, with five themes identified: (1) the nature and features of non-violent action, (2) the function of non-violent resistance, (3) motivations for non-violent resistance, (4) who or what the action opposed or made appeals to and (5) the specific forms of non-violent resistance. Results are summarised in Figure 1.

#### The nature and features of non-violent action

Most broadly, 44 (31%) participants identified non-violent resistance as an act or action. Ten (7%) participants identified that non-violent action often sought change of some type. A smaller number (*n* = 10, 7%) explicitly mentioned that such action could be carried out by individuals or groups and that action could involve acts of commission or omission (*n* = 2, 1%). As will be seen further on, many of these things were heavily implied elsewhere in other participants’ accounts of non-violent resistance. Looking more closely at the nature of non-violent action itself, an issue that weighed heavily related to harm or violence that came with action. Sixty six (47%) participants noted that such action should be peaceful or non-violent, while 19 (14%) felt that such action should not be ‘harmful’. Some participants called for the complete rejection of violence, while a smaller number felt that in some circumstances it could be justified. Some participants (*n* = 6, 4%) also made the point that non-violent resistance should also not be anti-social, while seven (5%) mentioned damage to property; two participants explicitly stated that non-violent resistance should not involve damage to property.

…absolutely no violence, not even resistance as the Police drag you away (Participant 41).

Resisting injustice and upholding human rights in ways that do not include physical violence against other humans/living creatures/the environment. This does not necessitate condemnation of various means of resistance (including possible use of physical force) by others directly experiencing significant and systematic oppression, e.g. people living under occupation or colonialism (Participant 141).

One participant was sceptical that any action could truly ever be non-violent.

Although I don’t think it really makes sense as a term as there is no such thing as non-violence. Everyone is violent, just some people don't see the violence they perpetuate. For example when people lie in the road to stop traffic they are relying on the police to protect them from angry drivers. The police will use violence or the threat of violence to protect those protesters lying in the road. The protesters might not be being violent themselves but they are using their position to get other people to be violent on their behalf (Participant 48).

Several other features of action were noted to a far lesser extent. Twelve (9%) participants identified that such action could be legal or illegal or involve non-engagement or refusal. Further features of non-violent resistance, such as action being a last resort, extra-institutional, a means to de-escalate otherwise violent situations, not resisting arrest, its association with historic figures and its public facing nature were all identified as features of non-violent resistance by ≤ 5 participants.

#### The function of non-violent resistance

Another prominent feature amongst participant understanding of non-violent resistance was what could be best labelled the function of non-violent resistance. Fifty four (39%) of participants stated that non-violent resistance was in some way oppositional, utilising a range of terms such as disruption, obstruction, challenging, subversion and nuisance. A smaller number of participants also saw such action as didactic or as a means to raise awareness (*n* = 22, 16%), while a similar number saw it as expressive or symbolic (*n* = 20, 14%), that is, as a way to communicate a grievance or call for change.

#### Motivations for non-violent resistance

Where participants identified motivations for non-violent resistance, conscience or an ethical or moral belief or principal were cited most commonly (*n* = 12, 9%) followed by political motivations (*n* = 5, 4%). Two (1%) participants stated that such action should be deliberate or wilful.

#### Who or what was opposed

Arguably the most diverse responses related to what or who non-violent resistance was opposed. While many participants implied that such action was opposed to ‘something’, for example, responses were only recorded where a more specific answer was given. These responses fit into three categories (1) what was being opposed, (2) who was being opposed and (3) the broad or specific issues that were opposed. In terms of what was being opposed, actions/activities/events or behaviours (*n* = 12, 9%), systems or processes (*n* = 4, 3%), law or policy or rules (*n* = 11, 8%), the ‘status quo’ (*n* = 1, 1%), power (*n* = 1, 1%) and norms (*n* = 1, 1%) were all identified. In terms of who was being opposed, government or authorities (n – 22, 16%), corporations, NGOs or institutions (*n* = 5, 4%) and individuals (*n* = 1, 1%) were all identified. Looking first at the broad issues, injustice or oppression (*n* = 18, 13%), harmful or damaging practices (*n* = 10, 7%), wrongdoing (or perceived wrongdoing; *n* = 4, 3%), rights abuses (*n* = 2, 1%), inequality (*n* = 1, 1%), undemocratic practices (*n* = 1, 1%) and unethical practices (*n* = 1, 1%) were all identified as particular targets of opposition. Finally, amongst the more specific issues raised, threats to health or healthcare (*n* = 5, 4%), the environment and climate change (*n* = 3, 2%), militarisation (*n* = 2, 1%) and immigration (*n* = 2, 1%) were all identified as more specific targets of opposition.

Action that resists a particular norm, which may be religious, cultural, role-stereotype, governmental, legal, and which therefore pushes or breaks a boundary imposed by people in a position of authority (Participant 129).

#### Forms of non-violent resistance

Several forms of non-violent action were also used by participants as examples to describe non-violent resistance. First, several more broad terms were utilised by participants; protest (*n* = 35, 25%), campaign (*n* = 7, 5%), direct action (*n* = 5, 4%), activism (*n*= 5, 4%) and advocacy (*n* = 7, 5%). More specific acts were also identified, the most common being blocking public areas or roads (or other activity that involved using one’s body; *n* = 15, 11%), demonstrations (*n* = 12, 9%), sit-ins (*n* = 11, 8%), marches (*n* = 9, 6%), letter writing or public statements (*n* = 8, 6%) civil disobedience (*n* = 7, 5%), strike or other industrial action (*n* = 6, 4%). A range of other actions were also identified by ≤ 5 participants, including boycotts, silence, giving out literature, posters, street stalls, picketing, petitions, providing refuge for undocumented migrants, lobbying the government or corporations, attending meetings, sanctions, twitter or email campaigns, legal action, hunger strikes, providing healthcare and other resistance within the workplace, comedic acts, public shaming, feigned or wilful ignorance, subversion, singing and vigils. Again, and like some of the issues above, what actions could be considered non-violent resistance were discussed by a number of participants. For example, many felt that for an action to be considered non-violent resistance, it had to occur outside of established institutional channels.

I see it as a form of political activism which aims to seek change without resorting to violence but which also sits outside of the institutionalised forms of seeking change e.g. appealing to government / petitions / legal action. Examples of non violent direct action include sit ins, obstructing roads or bridges, occupying buildings or offices etc (Participant 85).

Finally, while most participants identified public facing non-violent resistance, arguably most interesting were the examples provided that related to resistance that occurred in the workplace, actions which were largely individual, subversive and were not public facing.

Not referring patients to OVM [Overseas Visitor Manager, responsible for charging certain categories of migrants for NHS care]/not asking questions about status in the first place (willful ignorance), ignoring OVM queries/emails. Speaking to colleagues/encouraging them to do the same (Participant 78).

it covers multiple things and people can disagree about violent/non-violent [resistance]. But it might mean acts of disobedience/civil disobedience. Best examples that come to mind in healthcare are not doing Prevent training [counterterrorism awareness training which encourages health workers to report individuals suspected of ‘radicalisation’]; or actually leaving on time (Participant 96).

### Distinctions and differences in non-violent resistance

**Figure 2.**
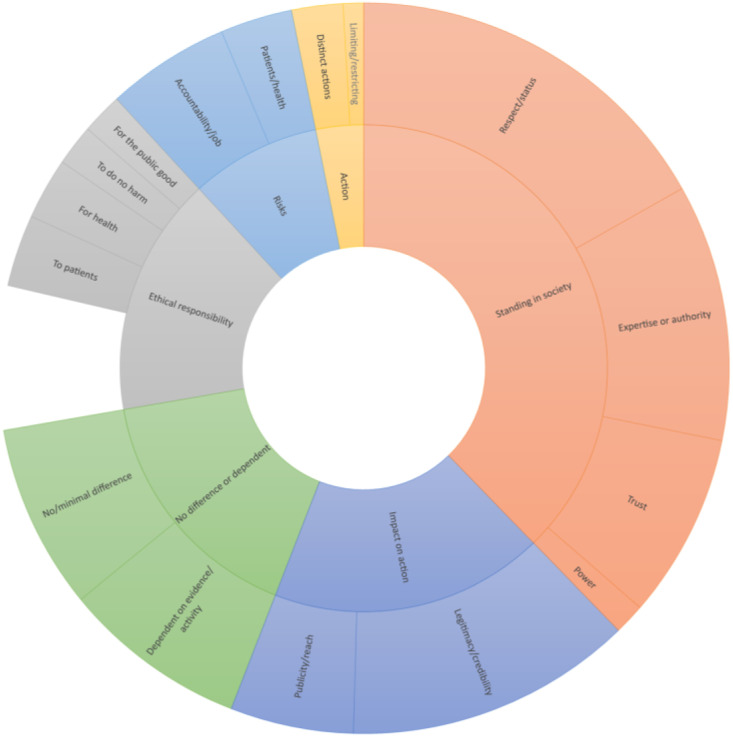
Sunburst chart of responses when asked about distinctions and differences in non-violent resistance when carried out by healthcare workers.

Building on the above question, participants were also asked if they felt that when healthcare workers engaged in non-violent resistance it made the action distinct or different in any way. Eighty-one participants (54.7%) said yes, 20 said no (13.5%), while 40 (27%) were unsure. One hundred and twenty two participants elaborated on why they felt this was the case. Responses fit into four themes: (1) the impact of healthcare workers on the action itself, (2) the moral or ethical responsibility for the action, (3) the risks that came with non-violent resistance and (4) that such action was either no different or largely dependent on the activity or issue in question. Results are summarised in Figure 2.

#### The impact of healthcare workers on non-violent action

Participants first and foremost felt their involvement in non-violent resistance could bolster the legitimacy or credibility of action (*n* = 28, 23%) and the publicity and reach (*n* = 12, 10%) of such action. Five participants (4%) identified that healthcare workers could engage in distinct actions because of their role, while two (2%) of participants identified that being a healthcare worker was in some way restrictive on the action they could engage in.

Identifying as hc [health care] workers means we have to be extra careful in chosen actions; e.g any that cause resultant harm or perceived harm to health as a consequence of actions will likely be detrimental to the cause (Participant 13).

I feel that doctors are expected to be "model citizens" and so shouldn't participate in non violent or violent resistance (Participant 32).

Health workers and others involved in the delivery of care know first hand the effects of cuts and destabilisation of services. They have the knowledge through experience to let others know of the impact of attacks on health care funding and resources (Participant 91).

A substantial number of participants identified that whether restrictive or not, the actions they could take were largely due to their standing in society. A number of participants mentioned healthcare workers’ respect or status in society (*n* = 37, 30%), their relative power (*n* = 3, 2%) and their expertise or authority (*n* = 25, 20%) particularly about issues related to health. Eighteen participants (15%) also mentioned the trust that the public has for healthcare workers.

“doctors against…” is different to “we’re against…” “as a doctor…” is different to “my opinion is…” (Participant 82).

It depends upon the action, but often as public figures healthcare workers involvement can legitimise certain non violent resistance which can be dismissed as ‘a mob’ by those in power. Healthcare workers are often seen as acting in the interests of public health, although motivations are often the same for civil society, this can be a powerful message (Participant 103).

In discussing trust, a number of participants acknowledged that engagement in non-violent resistance could impact trust in a number of ways. The trust of healthcare workers from the general public may in some way lend legitimacy to the action in question; however, trust may also be negatively impacted by such actions.

Healthcare workers are trained, bound by codes of conduct and regularly participate in discussions regarding what is justified and moral. We therefore have an important reputation in society. Non-violent action is most often a form of transgression in the service of a higher value. There is a reciprocal relationship between the reputation of healthcare workers and the action in question, that may enhance or denigrate either or both (Participant 129).

A number of participants were sceptical themselves that healthcare workers were in any way special or held any special status, however acknowledged that the general public often held healthcare workers in high regard.

I think involvement of healthcare workers in NVA [non-violent action] is only distinctive to the extent that public opinion perceives it as such because of the esteem in which these professions may be held. This could make it harder for the targets of NVA to dismiss the action or use force in response (Participant 39).

I do not think there is anything ‘special’ or ‘remarkable’ about healthcare workers but I do recognise that some people and parts of society may perceive it as important. Particularly how the NHS has most recently been held up as so vital to national survival [during the COVID-19 pandemic] (Participant 43).

#### Moral or ethical responsibility

Many participants noted that action was distinct as healthcare workers had an ethical or moral responsibility. This was framed in a number of ways, as a responsibility for health (*n* = 6, 5%), for the public (*n* = 4, 3%), to protect against or do no harm (*n* = 4, 3%) or more specifically for their patients (*n* = 7, 6%). Fourteen (11%) participants indicated they felt that healthcare workers had a more general ethical responsibility either to engage in non-violent resistance or to oppose policy that harmed health, for example, amongst the many other things that such action could be opposed to.

… healthcare workers are governed by codes of conduct, of varying degrees of strictness, imposed by their regulatory bodies. These can either be interpreted as prohibiting NVDA [non-violent direct action], or as giving justification for it, depending on the circumstances (Participant 68).

#### Risks related to non-violent resistance

Several participants also identified several risks that came with non-violent resistance. Twelve (10%) participants recognised that non-violent resistance could potentially impact either their professional registration or more generally the standing of the healthcare workers. Seven (6%) participants identified potential risks to patient care if engaging in non-violent resistance

on a societal level, healthcare workers are held to higher forms of accountability and risk more if they are involved and therefore their involvement carries greater weight (Participant 85).

#### No difference or context dependent difference

Finally, a number of participants simply stated that their was either minimal or no difference between healthcare professionals and others engaging in non-violent resistance (*n* = 18, 15%), while eighteen (15%) participants felt that it was largely context dependent as to whether action was in some way distinct of different to other instances of non-violent resistance.

On the one hand, people's occupational roles might be fairly irrelevant to collective actions/activism…Alternately, and especially in the context of health related resistances or solidarity action with certain groups eg disability rights activists or service users, refusers or survivors, identifying as a healthcare worker can add power/impact to the action (Participant 72).

Healthcare workers are one part of the general people/community that undertake non-violent resistance. Their presence can often 'validate' acts of resistance as healthcare workers are seen as aligned with the best interests of the public/public health. The action isn't necessarily distinct because of their presence, but their presence may be used strategically to shift narratives and help the action succeed (Participant 107).

## Discussion

This study sought to explore understandings of non-violent resistance and whether healthcare workers made a distinct contribution to such action. Our results, like the literature more generally, suggest that while the concept has a number of relatively uncontested features, it also has a number of contested elements. We will discuss our results below in light of the broader literature.

When examining participant understanding of non-violent resistance, our participants generally referred to public, organised acts, that often broke the law. This understanding is similar to many dominant and popular definitions, for example, ‘the application of unarmed civilian power using non-violent methods such as protests, strikes, boycotts and demonstrations, without using or threatening physical harm against the opponent’.^24^ Notably, while not widespread, participants did identify a number of subversive acts that could be considered everyday resistance. Ignorance, both active and passive in the workplace was one such strategy; ignorance of patients’ migration status and in actively avoiding colleagues whose role is to identify and charge patients without documentation. Refusal was another, not doing certain elements of training in the workplace. Humour was mentioned, as was a form of action which had synergies with a work-to-rule strike or Scott’s^14^ identification of ‘foot dragging’, with one participant identifying that leaving on time could be an act of resistance.

Looking at the functions of resistance identified by participants, namely its oppositional, didactic and expressive or symbolic functions sit comfortably with how a number of theorists have conceptualised resistance, not only as oppositional, but as a creative, productive force.^25^ For Hayward and Schuilenburg^11^ resistance serves as a ‘solvent of doxa, to continuously question obviousness and common sense, in order to create a new image of thought, and thus to remind us that things do not have to stay the way they are’. Similarly, for Flohr^26^ resistance ‘occupies a threshold between contemporary configurations of power and the possibility that things might be otherwise’. Lilja^25^ argues that through repetition and over time, resistance can (and has) ‘produce new and emerging realities’, new ways of thinking, new ways of viewing old problems while promoting a different and better vision for the future.

In addition to the issues above, a number of participants did touch on some of the conceptual controversies that surround non-violent resistance. For example, the idea that resistors need to be acting with intent was touched upon by a small number of participants. There has been substantial debate about this in the literature, which is beyond the scope of this article; however, it should be said that whether an individual needs to know they are engaged in an act of resistance remains a point of contestation.^27^ There was also discussion about violence and where to draw the line regarding ‘non-violence’. This again mirrors discussions in the literature which have called into question the violent-non-violent dichotomy.^28^

Beyond the nature of non-violent resistance itself, participants were also asked if they felt non-violent resistance when carried out by healthcare workers was distinct in any way. We are unaware of any studies that have asked this or similar questions. Overall, while opinion was divided, participants felt that healthcare workers could bolster the legitimacy or credibility of action and increase its publicity or reach. Participants felt this was due to the status they were aware their professions had in society and their relative authority, particularly in relation to issues related to health. Participants saw this working both ways, both as a factor which could limit action, for example, if an action risked damaging public trust, but also as a factor which allowed them, at times, to leverage this relative power. Importantly on this point, a number of participants didn’t see this as being significant at all, or only in as far as the public perceived healthcare workers as being trusted or an authority on an issue. A second issue related to the risks that came with non-violent resistance. This included concerns about professional registration and even the views of their colleagues. Another notable difference was that perhaps because of these factors, such as authority and public trust, along with the fact that many healthcare workers had codes of ethics or professional standards to uphold, consistent with past studies ^21^ many felt that they had a moral responsibility to engage in non-violent resistance. Finally, a number of participants felt that whether action was distinct was dependent on the action itself and often related to how closely the issue or action aligned with health.

While our participants brought with them a depth of knowledge and experience related to resistance that is unlikely to be found amongst healthcare workers, it is noteworthy in that this is also a limitation of this study, namely, that these findings may not be generalisable to other healthcare workers or settings. It is safe to assume our sample engaged in acts of non-violent resistance far more frequently than we might expect other healthcare professionals and were thus more sympathetic to such action. Our results are also not reflective of the broader healthcare workforce in the UK in relation to proportion of professionals we would expect to respond; nurses were underrepresented in our survey results. Furthermore, while we did not ask about political views or affiliations, given the population from which our participants were recruited, it is likely that most held relatively progressive political views, in this respect we did potentially miss other forms of resistance; there is a small body of work which outlines acts of resistance carried out by healthcare professionals that many may consider misguided or even harmful.^29^ On this point, this study also makes no normative claims about resistance, this is for another time. Finally, this study is also limited in that we did not seek to examine resistance within and between professions, for example, nursing and medical staff.^21^ While these issues did reveal themselves in some cases, for example, where participants refused to cooperate with hospital administrators in relation to determining eligibility for care, we did not seek to document these differences, this issue deserves far more attention than what it can be given here.

## Conclusion

Our findings speak to the complex and multifaceted nature of non-violent resistance. Further to this our findings suggest that healthcare workers have a distinct role to play in leading and supporting non-violent actions, especially at a time when health workers are in the public eye and governments are seeking to inhibit various forms of protest.^30^ Future research should explore these issues, but also several other questions raised above including how resistance could serve as oppositional, didactic and expressive along with exploring what makes such action impactful. Importantly, we hope this leads to a greater shared understanding of what resistance is, its core and more disputed features. We also hope this leads at least partly to having a shared vocabulary when approaching these issues and a foundation on which further discussion can be built. Beyond this article, we have said little about a range of other issues, the normative and regulatory concerns resistance raises, for example. These questions remain particularly pressing, given that in the UK, for example, regulatory bodies have been deliberately vague in their statements about whether healthcare workers will face disciplinary action if engaging in non-violent resistance.^31^ These findings should also be used as a foundation to begin to explore normative issues related to non-violent resistance. For example, should nurses be involved in non-violent actions? What forms of resistance in the workplace are justified? These are important questions and will be increasingly pressing for nursing ethics and professional regulatory bodies. We hope more broadly, that these findings begin a conversation not only about what non-violent resistance is but also about the involvement of healthcare workers in such action, and the potential that such action has in securing and protecting health and wellbeing.

## Supplemental Material

Supplemental Material - Exploring the concept of non-violent resistance amongst healthcare workers Click here for additional data file. Supplemental Material for Exploring the concept of non-violent resistance amongst healthcare workers by Ryan Essex, Hil Aked, Rebecca Daniels, Paul Newton and Sharon Weldon in Nursing Ethics
