# Supplementary material for: Exploring the concept of non-violent resistance amongst healthcare workers
Source: Nurs Ethics. 2022 Oct 6;30(1):7–19. doi: 10.1177/09697330221122904 (PMC9902996; doi:10.1177/09697330221122904)
Supplement: Supplemental Material - Exploring the concept of non-violent resistance amongst healthcare workers [file sj-pdf-1-nej-10.1177_09697330221122904.pdf]

Supplementary material for reviewers only – survey – adapted from Qualtrics

---

What do you think is meant by non-violent resistance/action?

For the remainder of this survey, when we refer to non-violent resistance/action we mean it as an umbrella term to refer to a range of political actions. Such action could be organised and public or individual and carried out in private. Non-violent resistance can undertaken in response to a range of issues and can also be legal or illegal. Such action could include "protests", "marches", "lobbying", "flyering/leafleting", "whistleblowing", "civil disobedience" and even "everyday acts" performed in your day to day activities or in your role as a healthcare worker.

Does the involvement of healthcare workers in non-violent action make the action distinct or different in any way?

Yes	No	Unsure
◯	◯	◯

Please elaborate **(THIS QUESTION WILL BE OPTIONAL)**

Do you have any further thoughts or concerns about non-violent resistance that you would like to

leave here? **(THIS QUESTION WILL BE OPTIONAL)**

____

Demographic information

We would like to know a little more about you, however all of the following questions are optional.

Are you retired?

Yes	No
◯	◯

Have you ever engaged in non-violent resistance? (for these purposes we mean all forms of action from civil disobedience, marches, lobbying, flyering/leafleting etc)

Yes	No
◯	◯

If so, how often do you generally engage in such action?

Less than once a year	About once a year	A few times a year	About every month	A few times every month	About every week or more
◯	◯	◯	◯	◯	◯

What type of non-violent resistance/action have you engaged in (please tick all that apply)?

1. Civil disobedience	◯
2. Strike action	◯
3. Marches	◯
4. Everyday acts at home/at work	◯
5. Whistleblowing	◯
6. Lobbying	◯
7. Vigils	◯
8. Sit-ins	◯
9. Online activism	◯
10. Flyering/leafleting	◯
Other	◯

If other, please specify

What is your age?

18-24	25-34	35-44	45-54	55-64	65+
◯	◯	◯	◯	◯	◯

Gender

Male	Female	Other
◯	◯	◯

What best describes your profession now or prior to your retirement?

Doctor	◯
Nurse	◯
Midwife	◯
Occupational therapist	◯
Psychologist	◯
Paramedic	◯
Physiotherapist	◯
Radiographer	◯
Academic	◯
Other	◯

If other, please specify

In your role in healthcare what level of seniority do you hold, now or prior to your retirement?

Junior	Mid-level	Senior
◯	◯	◯

Race/Ethnicity

Asian/Asian British	◯
Black/African/Caribbean/Black British	◯
Mixed/Multiple ethnic groups	◯
White	◯
Other	◯

If other, please specify
